# The Herald of Health

**Published:** 1876-01

**Authors:** 


*The Herald of Health*, devoted to the culture of the Body and Mind Publishers: Wood & Holbrook, 13 and 15 Laight Street, N. Y. Terms: $2.00 per annum with premium; $1.50 without.

This is one of the most popular health journals ever published, containing matter which intereste all classes of society, and proves useful in many ways to its readers. This *Journal* has now reached its 25th volume of the new series, after an old series of 59 volumes; is printed in large clear type and admirably conducted. Wood & Holbrook are also the publishers of a small, but very interesting and useful work, entitled "Eating for Strength," a new health cookery book, edited by M. L. Holbrook, M. D., which gives every necessary direction for the preparation of simple, wholesome food, with little labor.

